# Comparison of surgical approaches to the hippocampal formation with artificial intelligence

**DOI:** 10.1007/s10143-025-03345-z

**Published:** 2025-02-19

**Authors:** Tolga Turan Dundar, Meltem Kurt Pehlivanoğlu, Ayşe Gül Eker, Nur Banu Albayrak, Ahmet Serdar Mutluer, İsmail Yurtsever, İhsan Doğan, Nevcihan Duru, Uğur Türe

**Affiliations:** 1 Department of Neurosurgery, Bezmialem Vakif University, Istanbul, Turkey; 2 Department of Biostatistics and Medical Informatics, Istanbul University-Cerrahpasa, Istanbul, Turkey; 3 Faculty of Computer Engineering, Kocaeli University, Kocaeli, Turkey; 4 Faculty of Engineering and Natural Sciences, Kocaeli Health and Technology University, Kocaeli, Turkey; 5 Faculty of Medicine, Bezmialem Vakif University, Istanbul, Turkey; 6 Department of Radiology, Bezmialem Vakif University, Istanbul, Turkey; 7 Department of Neurosurgery, Ankara University, Ankara, Turkey; 8 Department of Neurosurgery, Yeditepe University School of Medicine, Kosuyolu Mah. Kosuyolu Cad. No: 168, Kadikoy, Istanbul, 34718 Turkey

**Keywords:** Artificial intelligence, Cranial approaches, Machine learning, Mediobasal temporal region, Neurosurgical planning

## Abstract

The relatively complex functional anatomy of the mediobasal temporal region makes surgical approaches to this area challenging. Several studies describe various surgical approaches, along with their combinations and modifications, to reach lesions of this region. Some of these surgical approaches have been compared using artificial intelligence-based approaches that can be predicted, classified, and analyzed for complex data. Several surgical approaches, such as anterior transsylvian, trans-superior temporal sulcus, trans-middle temporal gyrus, subtemporal–transparahippocampal, presigmoid-retrolabyrinthine, supratentorial-infraoccipital, and paramedian supracerebellar-transtentorial, were selected for comparison. Magnetic resonance images (MRIs) were taken according to the criteria specified by the Radiology Department. With an open-source software tool, volumetric data from cranial MRIs were segmented and anatomical structures in the main regions were reconstructed. The Q-learning algorithm was used to find pathways similar to these standard surgical pathways. The Q-learning scores among the selected pathways are as follows: anterior transsylvian (Q_A) = 31.01, trans-superior temporal sulcus (Q_B) = 25.00, trans-middle temporal gyrus (Q_C) = 28.92, subtemporal-transparahippocampal (Q_D) = 23.51, presigmoid- retrolabyrinthine (Q_E) = 27.54, supratentorial-infraoccipital (Q_F) = 27.2, and paramedian supracerebellar-transtentorial (Q _G) = 21.04. The Q-value score for the supracerebellar transtentorial approach was the highest among the examined approaches and therefore optimal. A difference was also found between the total risk score of all points with pathways drawn by clinicians and the total risk scores of the pathways formed and followed by Q-learning. Artificial intelligence-based approaches may significantly contribute to the success of the surgical approaches examined. Furthermore, artificial intelligence can contribute to clinical outcomes in both preoperative surgical planning and intraoperative technical equipment-assisted neurosurgery. However, further studies with more detailed data are needed for more sensitive results.

## Introduction

**Fig. 1:**
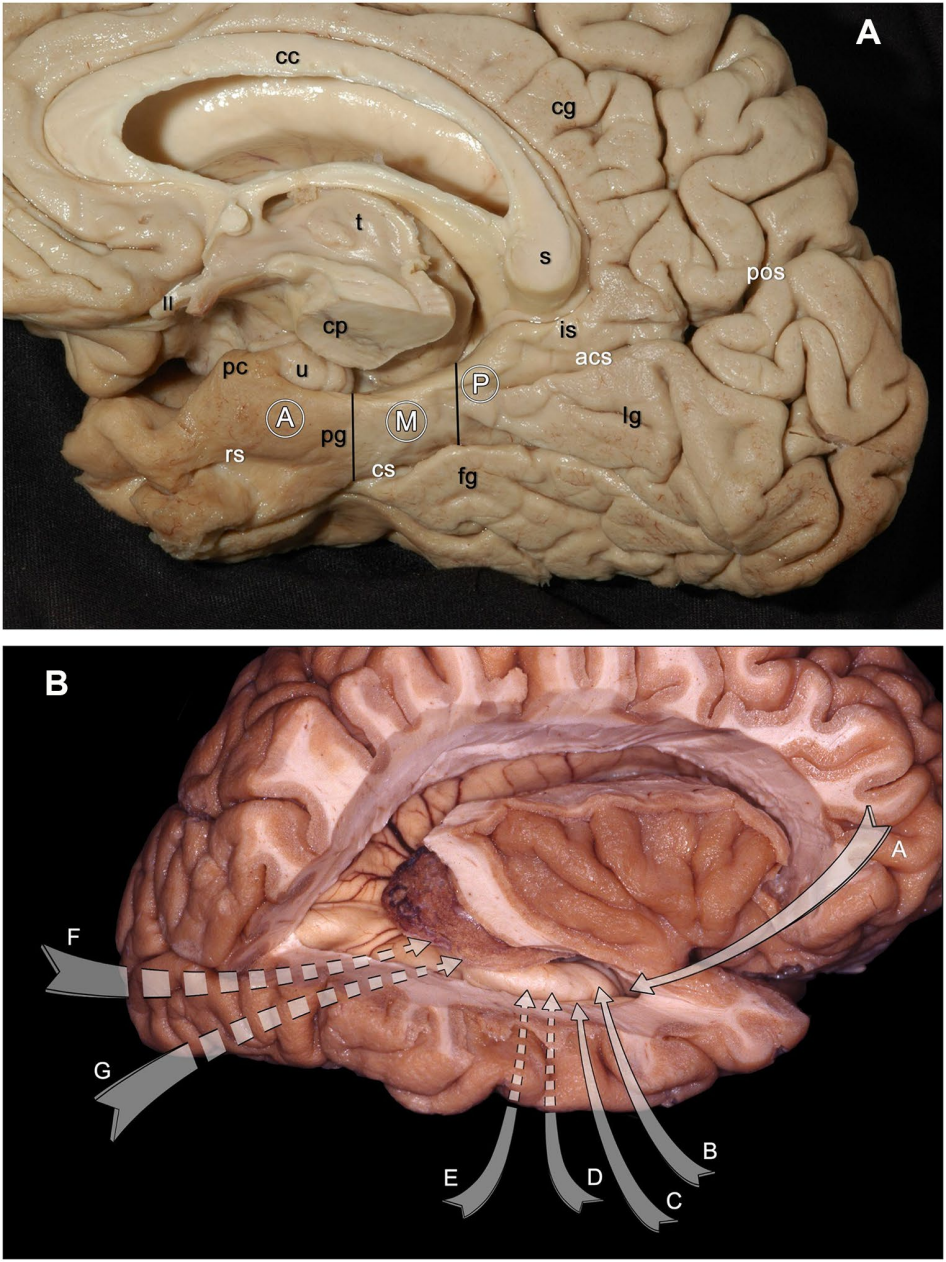
(**A**) Inferolateral view of the MTR in the right cerebral hemisphere after the insula and the lateral and third ventricles were exposed and the corona radiata was cut in a cadaver specimen. The MTR is divided into 3 portions: anterior, middle, and posterior. The anterior portion begins at the piriform cortex and extends to the tip of the uncus. The temporal incisura and the rhinal and collateral sulci form the lateral border of the anterior portion. The amygdala, uncus, hippocampus, and the head of the parahippocampal gyrus form the anterior portion. The middle portion extends to the collicular level of the midbrain, and the collateral sulcus is the lateral border. The posterior portion of the MTR extends posteriorly from this level and divides into superior and inferior parts, as the anterior and calcarine sulcus separates these structures naturally. No exact border shows where the MTR ends. (**B**) Photograph showing a right cerebral hemisphere after partial removal of the frontal, temporal, and parietal lobes. The different surgical approaches are demonstrated with arrows. (**A**) anterior transsylvian approach; (**B**) trans-superior temporal sulcus approach; (**C**) trans- middle temporal gyrus approach; (**D**) subtemporal-transparahippocampal approach; (**E**) presigmoid-retrolabyrinthine approach; (**F**) supratentorial-infraoccipital approach; (**G**) paramedian supracerebellar transtentorial approach. Abbreviations with white letters denote the sulci. Ⓐ=anterior portion of the MTR; cc=corpus callosum; cg=cingulate gyrus; cp=cerebral peduncle; cs=collateral sulcus; fg=fusiform gyrus; lg=inferior gyrus; is=isthmus; Ⓜ=middle portion of the MTR; Ⓟ=posterior portion of the MTR; pos=parieto-occipital sulcus; pc=piriform cortex; rs=rhinal sulcus; s=splenium of the corpus callosum; t=thalamus; u=uncus; II=optic nerve

The inferomedial part of the temporal lobe is known as the mediobasal temporal region (MTR) and is a part of the limbic system [1, 2]. This area is a common target location for neurosurgical procedures to treat epilepsy, tumors, cavernomas, and arteriovenous malformations, among other pathologies. The MTR is limited medially by the lateral wall of the cavernous sinus and the carotid, crural, and ambient cisterns. It is bounded laterally by the collateral and rhinal sulci and anteriorly by the lesser wing of the sphenoid bone. The tip of the cuneus, which is the isthmus of the cingulate gyrus and the connection between the parieto-occipital and the calcarine sulci, forms the posterior border of the MTR [3]. Nevertheless, the MTR is divided into three portions: anterior, middle, and posterior. The area from the piriform cortex to the tip of the uncus is the anterior portion. The temporal incisura and the rhinal and collateral sulci form the lateral border of the anterior portion. This portion consists of the amygdala, uncus, head of the parahippocampal gyrus, and hippocampus. The middle portion extends to the collicular level of the midbrain. The collateral sulcus is the lateral border of the middle portion of the MTR. The posterior portion extends posteriorly from this area [3, 4] (Fig. 1A, B). (Table 1). They contain vascular and neural structures that must be preserved during surgery [1, 5, 6].

**Table 1:** Anatomic structures in and around the mediobasal temporal region

Mediobasal Temporal Region	
**Neural Tissue**	Optic tract Lateral geniculate body Optic radiation Oculomotor nerve Trochlear nerve Midbrain Basal language area in the fusiform gyrus Anterior commissure Uncinate fasciculus
**White Matter of Temporal Lobe**	1. Short association (U) fibers 2. Superior longitudinal fasciculus (inf. arm) 3. Uncinate fasciculus 4. Occipitofrontal fasciculus 5. Anterior commissure 6. Sublenticular part of the internal capsule I. Temporopontine fibers II. Occipitopontine fibers III. Inferior thalamic peduncle IV. Posterior thalamic peduncle (inc. auditory and optic radiations) 7. Tapetum 8. Inferior longitudinal fasciculus 9. Alveus-fimbria-fornix 10. Cingulum (inferior arm)
**Vascular Structures**	MCA AChA PCA Superficial and deep Sylvian veins Temporal basal veins Basal vein of Rosenthal Vein of Labbé

**Fig. 2:**
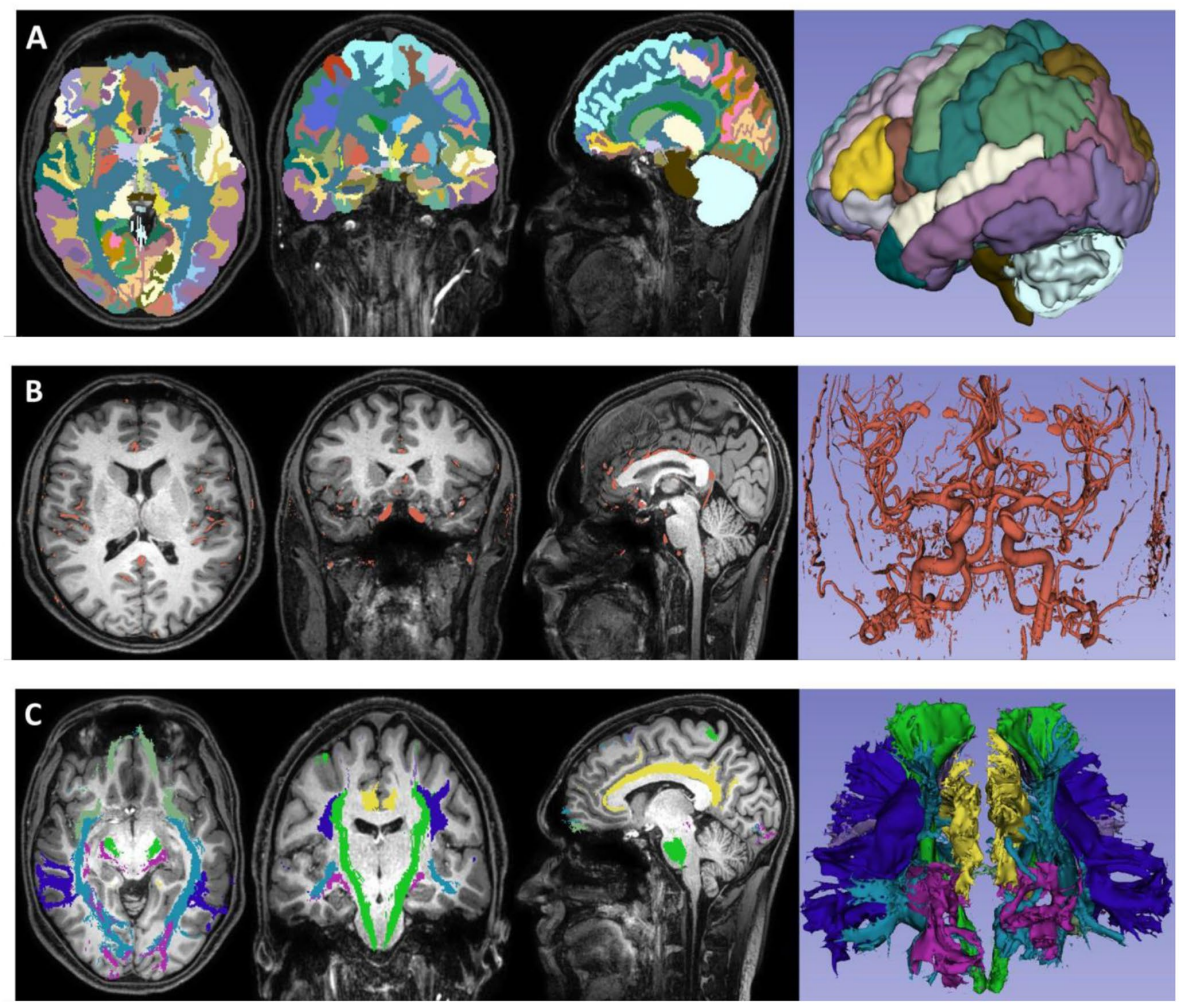
The extracted segments, vascular fields, and main tracts of the chosen sample from the cohort. This picture was obtained by segmenting the MRI images taken by the radiology department through the program. The anatomical areas in these MRI scans were evaluated using the coordinate system. (**A**) segmented brain image; (**B**) extraction of the vascular anatomy; (**C**) reconstruction of the main tracts

Surgeons must choose the surgical approach to the MTR carefully because of the distinct and relatively complex anatomy of this area. The literature has described a variety of methods and variations for safe surgery to access the MTR (Table 1). However, each surgical technique inevitably results in unpredictable and avoidable trauma to the tissue in the immediate vicinity [4, 7, 8]. Despite many successful results reported in surgical series, there is no consensus on the ideal surgical approach [9, 10].

Neurosurgery, as with all medical fields, is affected by advances in technology and science. Similar to other medical fields, neurosurgery has seen a sharp rise in interest in the applications of artificial intelligence (AI) [10, 11]. AI refers to computer systems that can replicate certain cognitive functions of the human brain, including learning, reasoning, and self-correction. This automatic learning and understanding of vast amounts of data, which results in knowledge and experience-based action, is a capability of AI. There are several types of machine learning (ML), including supervised learning, unsupervised learning, semi-supervised learning, and reinforcement learning. Q-learning (QL) is a popular algorithm of reinforcement learning [10, 12]. It does this using a Q-table to store the expected future reward for taking a particular action in a given state and updating the table over time based on the observed outcomes of those actions. The algorithm uses equation to update the Q-values and eventually finds the optimal policy by selecting the action with the highest Q-value in each state.

The retrocomissural portion of the hippocampal formation, which can be very difficult to approach in the MTR, was chosen as the target area. Our study compared the commonly used surgical approaches to the MTR with a QL algorithm.

## Materials and methods

A number of the many approaches to the MTR described in the literature were compared. These included the anterior transsylvian, trans-superior temporal sulcus, trans-middle temporal gyrus, subtemporal-transparahippocampal, presigmoid-retrolabyrinthine, supratentorial-infraoccipital, and paramedian supracerebellar-transtentorial approaches.

### Dataset

The EDEN2020 Human Brain MRI Datasets for Healthy Volunteers dataset, which consists of high-resolution MRIs of 15 healthy adults, was the starting point [13, 14]. From this dataset, T1_3D_PROSET_Sag, MIP_s3DI_MC_HR, and raw_data_NODDI data were utilized to obtain T1-weighted (T1) volumetric sequences, time-of-flight magnetic resonance angiography (TOF-MRA), and diffusion tensor imaging (DTI) tractography reconstructions, respectively. For the visualization and comparison of different surgical pathways, T1, TOF-MRA, and DTI data for just one adult from the cohort were selected (as seen in the sample CTRL_1277.zip in the dataset). MRI data was taken according to the specified criteria were also utilized by the Radiology Department. In the example provided, the 3D MRI is segmented based on the T1 sequences, and its dimensions are 320 × 320 × 236. The total number of voxels is 24,166,400. The image spacing is 0.8 mm, which indicates the physical distance between the centers of adjacent pixels in each dimension (X, Y, and Z). Each pixel is separated by 0.8 mm in the X and Y directions, and each slice (Z direction) is also spaced by 0.8 mm. In 3D Slicer, the Registration tool has been used for the fusion of images from different modalities. This tool is a comprehensive set of tools designed to support registration or image fusion tasks. Using this tool, all images are fused, and at corresponding points that have different scores, the highest penalty score is assigned as valid.

### Segmenting the brain

To obtain volumetric data, the brain was segmented into main regions using the BrainSuite tool [14] with data was obtained from T1 sequences. A high-resolution single-subject atlas, the USC Brain Atlas [15], was chosen for parcellation of the cortex. The 3D Slicer tool [16] was used to extract the vascular anatomy from TOF-MRA data.

### Reconstructing anatomical structures

**Fig. 3:**
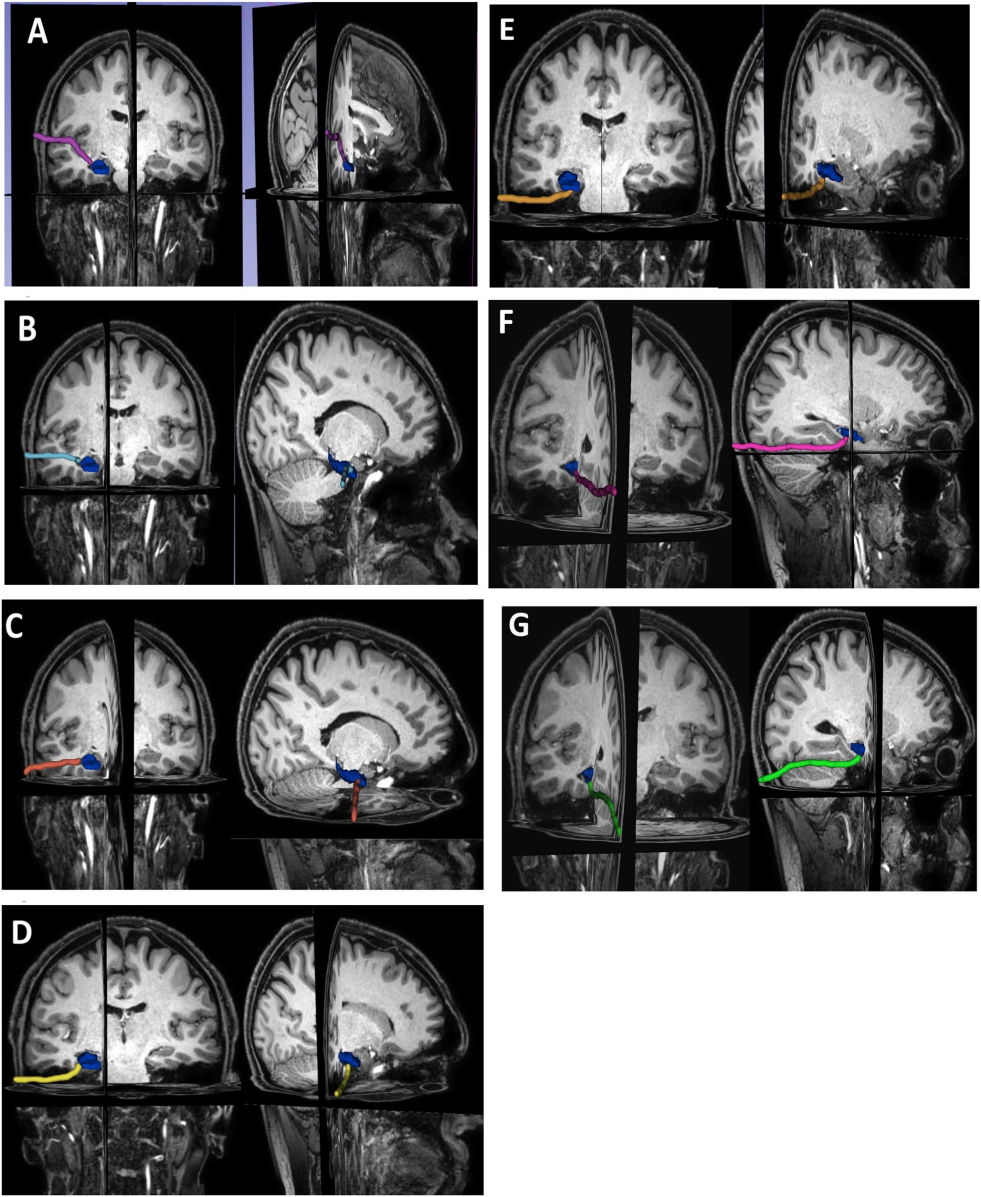
The hand-drawn pathways for the chosen sample from the cohort. (**A**) anterior transsylvian; (**B**) trans-superior temporal sulcus; (**C**) trans-middle temporal gyrus; (**D**) subtemporal-transparahippocampal; (**E**) presigmoid-retrolabyrinthine; (**F**) supratentorial-infraoccipital; (**G**) paramedian supracerebellar-transtentorial

Using the 3D Slicer tool, all the fiber tracts given in the DTI data were masked. Figure 2 displays the segments, vascular fields, and main tracts of the sample data. By combining these factors, a 3D map of the brain was created using the 3D Slicer; penalty risk scores were then assigned for each one. While a score of 1 is defined as a penalty, a score of 0 indicates no penalty. All segmented fields were scored by three clinicians and the scores were averaged out and given in Table 2. Thus, each coordinate was assigned a point value with the so-called risk score. The clinicians drew the existing selected pathways (anterior transsylvian, trans-superior temporal sulcus, trans-middle temporal gyrus, subtemporal-transparahippocampal, presigmoid-retrolabyrinthine, supratentorial-infraoccipital, and paramedian supracerebellar-transtentorial) using the Segment Editor Module [17] within the 3D Slicer tool for the MRI of the chosen sample (Fig. 2A, B and C). Figure 3A and B, and 3C show that the diameter of each pathway was chosen to be 0.4 cm [10, 18]. Note that the drawings were created on a single MRI sequence of the chosen sample since the editor automatically matches the corresponding locations on the remaining MRIs of the chosen sample. The drawn pathways were converted into segments and saved as NRRD files. Then, all coordinates corresponding to pathways in each file were saved, and these coordinates were used to calculate the risk score of each one. The comparison of total risk scores for all selected pathways appears in Table 3. All risk scores were calculated by adding up the tract, vascular, and whole-brain segmentation field risk scores. After that, using the QL algorithm, we tried to find several new nonlinear surgical pathways similar to the existing seven (standard) ones that reach the MTR. It’s worth noting that clinicians solely determined the entry points for the newly extracted surgical pathways, with the QL algorithm responsible for extracting the complete pathways. In the search process, we create a Q-Table (Q-Matrix). This table is the main structure used in Q-learning, typically containing a Q-value for each stateaction pair. However, our Q-Table houses connection values between adjacent nodes instead of actions. The Q-Table is usually initially filled with random numbers. In our case, we fill the table with a random number if there is a connection between two nodes; otherwise, we fill it with − 1, indicating that there is no transition between those nodes. As the search progresses and a path is found, i.e., reaching the destination, the Q values in the table are updated. The update is performed using the Bellman equation (see Eq. 1). This equation ensures the updating of Q values, combining them with new information to obtain a more current prediction.

**Table 2:** Table of rated risk score of anatomical segments

Segment Name	Risk Score	Segment Name	Risk Score	Segment Name	Risk Score	Segment Name	Risk Score	Segment Name	Risk Score	Segment Name	Risk Score
R. globus pallidus	1.0	lingual gyrus- posterior	0.5	superior temporal gyrus- middle	0.6	pars opercularis- superior	1.0	paracentral lobule	1.0	cuneus- posterior	0.3
L. globus pallidus	1.0	cuneus - anterior	0.7	superior temporal gyrus- posterior	0.7	pars opercularis- inferior	1.0	cingulate gyrus- anterior	0.3	insula- posterior	1.0
R. caudate nucleus	1.0	cuneus- posterior	0.7	transverse temporal gyrus	1.0	pars triangularis- anterior	1.0	cingulate gyrus- middle	0.3	**Tracts**	1.0
L. caudate nucleus	1.0	insula- anterior	1.0	middle temporal gyrus - anterior	0.5	pars triangularis- middle	1.0	cingulate gyrus- posterior	0.3	middle occipital gyrus- ventroanterior	1.0
R. putamen	1.0	insula- posterior	1.0	middle temporal gyrus-middle	0.5	pars triangularis- posterior	1.0	subcallosal gyrus	0.3	middle occipital gyrus- posterior	1.0
L. putamen	1.0	white matter (cerebrum)	-	middle temporal gyrus-dorsoposterior	0.7	pars orbitalis	1.0	postcentral gyrus- superior	1.0	inferior occipital gyrus-anterior	1.0
R. thalamus	1.0	superior frontal gyrus- anterior	0.3	middle temporal gyrus- ventroposterior	0.5	precentral gyrus-inferior	1.0	postcentral gyrus- inferior	1.0	inferior occipital gyrus- dorsoposterior	1.0
L. thalamus	1.0	superior frontal gyrus- posterior	1.0	inferior temporal gyrus-anterior	0.5	precentral gyrus- superior	1.0	supramarginal gyrus- anterior	1.0	inferior occipital gyrus- ventroposterior	1.0
R.nucleus accumbens	1.0	middle frontal gyrus- anterior	0.3	inferior temporal gyrus-anterior	0.5	transverse frontal gyrus- mesial	0.3	supramarginal gyrus- anterior	1.0	inferior occipital gyrus- ventroposterior	1.0
L.nucleus accumbens	1.0	middle frontal gyrus- posterior	1.0	inferior temporal gyrus-middle	0.5	transverse frontal gyrus-lateral	0.3	angular gyrus- anterior	1.0	lingual gyrus- anterior	0.5
R.superior colliculus	1.0	pars opercularis- superior	1.0	inferior temporal gyrus-posterior	0.5	gyrus rectus	0.3	angular gyrus- middle	1.0	lingual gyrus- posterior	0.5
L.superior colliculus	1.0	pars opercularis- superior	1.0	fusiform gyrus-anterior	0.3	middle orbital frontal gyrus	0.3	angular gyrus- posterior	1.0	postcentral gyrus-inferior	1.0
R.inferior colliculus	1.0	pars opercularis- inferior	1.0	fusiform gyrus-posterior	0.7	arithmetic orbital frontal gyrus	0.3	superior parietal gyrus- anterior	1.0	supramarginal gyrus-anterior	1.0
L.inferior colliculus	1.0	pars triangularis- anterior	1.0	perahippocampal gyrus	1.0	hippocampus	1.0	superior parietal gyrus- posterior	1.0	supramarginal gyrus-posterior	1.0
R. mammillary body	1.0	pars triangularis- middle	1.0	amygdala	0.5	lateral orbitofrontal gyrus- anterior	0.3	precuneus- superior	0.5	angular gyrus-superior	1.0
L. mammillary body	1.0	pars triangularis- posterior	1.0	superior occipital gyrus-superior	0.3	lateral orbitofrontal gyrus- posterior	0.3	precuneus-inferior	0.5	angular gyrus-middle	1.0
pineal gland	0.5	pars orbitalis	1.0	superior occipital gyrus - inferior	0.3	paracentral lobule	1.0	temporal pole	0.3	angular gyrus- posterior	1.0
R. lateral ventricle	0.1	precentral gyrus- inferior	1.0	middle occipital gyrus - dorsocentror	1.0	cingulate gyrus- anterior	0.7	superior temporal gyrus- anterior	0.3	superior parietal gyrus-anterior	0.5
L. lateral ventricle	0.1	precentral gyrus- superior	1.0	middle occipital gyrus- ventroanterior	1.0	cingulate gyrus- middle	0.7	superior temporal gyrus- middle	0.3	superior parietal gyrus- posterior	0.5
third ventricle	0.1	transverse frontal gyrus- lateral	0.6	superior occipital gyrus-inferior	0.7	cingulate gyrus- posterior	0.7	superior temporal gyrus- posterior	0.3	precuneus- superior	0.5
fourth ventricle	0.1	gyrus rectus	0.3	middle occipital gyrus- dorsocentror	0.7	subcallosal gyrus	0.5	transverse temporal gyrus	0.3	precuneus- inferior	0.5
cerebral aqueduct	0.1	middle orbitofrontal gyrus	0.3	middle occipital gyrus- ventroanterior	1.0	postcentral gyrus- superior	1.0	transverse temporal gyrus	0.5	temporal pole	0.3
Brainstem	1.0	anterior orbitofrontal gyrus	0.3	middle occipital gyrus-posterior	1.0	middle temporal gyrus- anterior	0.7	inferior temporal gyrus- anterior	0.7	fusiform gyrus- posterior	0.3
superior frontal gyrus- anterior	0.5	posterior cingulate gyrus	0.3	inferior occipital gyrus- anterior	1.0	middle temporal gyrus- middle	0.7	inferior temporal gyrus - middle	0.3	parahippocampal gyrus	0.3
superior frontal gyrus- posterior	1.0	lateral orbitofrontal gyrus - anterior	0.3	inferior occipital gyrus- dorsoposterior	1.0	middle temporal gyrus- dorsoposterior	0.7	inferior temporal gyrus- posterior	0.3	hippocampus	0.7
middle frontal gyrus- anterior	0.5	lateral orbitofrontal gyrus- posterior	0.3	inferior occipital gyrus- ventroposterior	1.0	middle temporal gyrus- ventroposterior	0.7	fusiform gyrus- anterior	0.3		
middle frontal gyrus- posterior	1.0	lateral orbitofrontal gyrus- posterior	0.3	lingual gyrus- posterior	0.3						

*The segmented fields were scored by two neurosurgeons (TD, ID) and one radiologist (IY)

**Table 3:** Total risk scores for all selected pathways*

Pathway	Total risk scores	Total Q-values	Total risk scores Qr
Anterior trans-sylvian	A = 137.8	Q_ A = 31.0138	Qr_ A = 116.39
Trans-superior temporal sulcus)	B = 79.20	Q_ B = 25.00	Qr_ B = 33.2
Trans- middle temporal gyrus)	C = 54.66	Q_ C = 28.929	Qr_ C = 56.3
Subtemporal- transparahippocampal)	D = 28.66	Q_ D = 23,51	Qr_ D = 82.4
Presigmoid- Transpetrosal)	E = 61.2	Q_ E = 27.54	Qr_ E = 58.7
Supratentorial-infraoccipital)	F = 31.70	Q_ F = 27.2	Qr_F = 32.7
Paramedian Supracerebellar Transtentorial)	G = 31.60	Q_ G = 21.048	Qr_ G = 32.4

*A comparison of the sum of the total risk scores (A-G) of each pathway was done by the same clinicians. The risk score was determined through the arithmetic addition of anatomic areas. The total Q-values and risk scores of surgical pathways were drawn with artificial intelligence, yielding total Q-values and risk scores of pathways Q_A, Q_B, Q_C, Q_D, Q_E, and Q_F. Here, Q-values give extra information to determine how good an action is. The lowest total Q-value defines the best pathway with the minimum penalty score. Qr represents the arithmetic sum of segment risk scores on the path drawn by artificial intelligence

*Q*(*S*,* A*) = *Q*(*S*,* A*) + *α*[*R* + *γ* argmax*Q*(*S*^′^,*A*^′^) − *Q*(*S*,* A*)] (1)

In this equation, *A* is the current action, *S* is the current state, *γ* is the discount factor, *α* is the learning rate, *Q*(*S*,* A*) represents current Q values, and argmax*Q*(*S*^′^,*A*^′^) represents the best state-action pair in the next state. In certain scenarios, QL can also serve as a path-finding algorithm [19]. Therefore, in this paper, we incorporate risk scores into our approach and utilize the QL algorithm to uncover non-linear surgical pathways that minimize penalty scores.

**Fig. 4:**
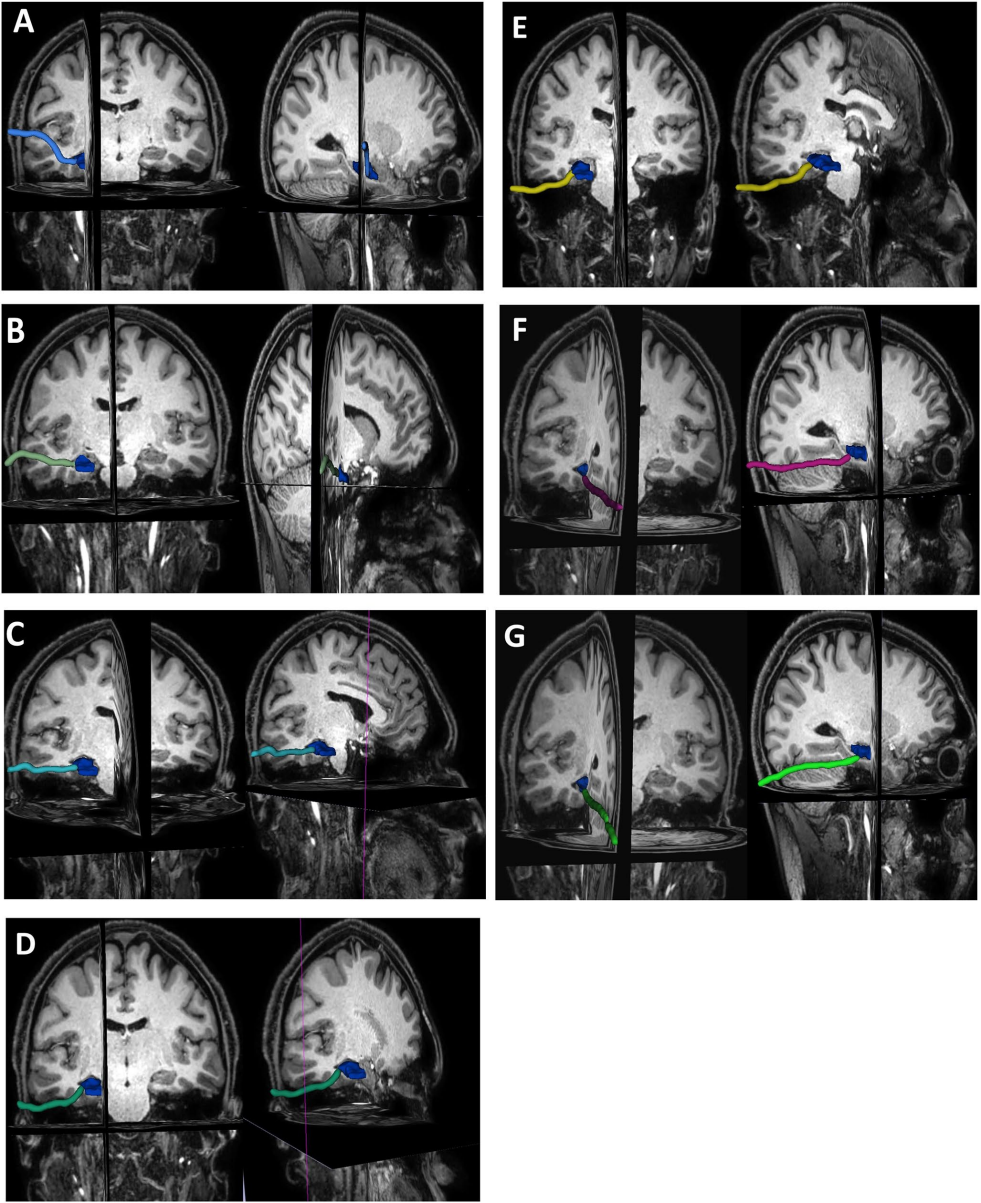
With Q learning, approaches are created with more location calculations

Once the search is completed after a certain number of epochs, and a path is found, the Q table takes its most up-to-date form. The nodes on the discovered path are presented as output. The Q-values of the nodes given as output between the starting and destination nodes in the Q table are summed to calculate the Q-learning score. In our structure, 0 represents risk-free structures, while 1 indicates risky structures. Each node has a value between 0 and 1. Therefore, the lower the Q-learning score we obtain, the more optimal the path is, meaning it is less risky. Finally, we successfully identified novel pathways (Q_A, Q_B, Q_C, Q_D, Q_E, Q_F, and Q_G) that resemble the standard ones. We computed the total Q-values (Qr_A, Qr_B, Qr_C, Qr_D, Qr_E, Qr_F, and Qr_G, which are derived from the summation of all Q-values along each pathway) and the respective risk scores. All of these computed values are displayed in Table 3, while Fig. 4 visually presents the newly discovered surgical pathways extracted using the QL algorithm.

## Results

First, the pathways were drawn on the MRI of the chosen sample. Then, all the risk scores of the paths were calculated using the score table (Table 2). The scores were as follows: anterior transsylvian (A) = 137.8, transsuperior temporal sulcus (B) = 79.2, trans-middle temporal gyrus (C) = 54.66, subtemporal-transparahippocampal (D) = 28.66, presigmoid- retrolabyrinthine (E) = 61.2, supratentorial-infraoccipital (F) = 31.7, and paramedian supracerebellar transtentorial (G) = 31.6. Thus, the paths with total low-risk scores contain low-risk areas.

Next, these scores were compared to the scores of new paths found with the QL algorithm (Table 3). The total Q-learning score of the new pathways were as follows: anterior transsylvian (Q_A) = 31.0138, trans-superior temporal sulcus (Q_B) = 25.00, trans-middle temporal gyrus (Q_C) = 28.929, subtemporal-transparahippocampal (Q_D) = 23.51, presigmoid-retrolabyrinthine (Q_E) = 27.54, supratentorial-infraoccipital (Q_F) = 27.2, and paramedian supracerebellar transtentorial (Q_G) = 21.04. These pathways were drawn by AI (Table 3).

For each path, these values were calculated by adding all the Q-learning score on the corresponding path. Essentially, the QL algorithm found all these paths by following each point and the best possibility after that point. Getting the lowest score from the QL algorithm gave the advantage to a specific approach.

Finally, the total risk scores for Qr(A, B, C, D, E, F, G) of each identified path were determined as follows: anterior transsylvian (Qr_A) = 116.39, trans-superior temporal sulcus (Qr_B) = 33.2, trans-middle gyral (Qr_C) = 56.3, subtemporal-transparahippocampal (Qr_D) = 82.4, presigmoid- retrolabyrinthine (Qr_E) = 58.7, supratentorial-infraoccipital (Qr_F) = 32.7, and paramedian supracerebellar transtentorial (Qr_G) = 32.4 (Table 3).

The algorithm employed in our study is noteworthy for its lack of any randomness elements, ensuring that its outcomes are both consistent and stable. When executed multiple times under identical conditions, the algorithm is designed to consistently select the same paths. This deterministic nature was particularly evident in our comparative analysis of surgical approaches. For instance, while the subtemporal approach received a reasonable risk score, determined by arithmetically summing the anatomical areas identified by the reviewers, it was the supracerebellar transtentorial approach that attained the optimal Q-learning score. This consistency in algorithmic decision-making underlines the reliability of our method in selecting the most appropriate surgical pathways, further emphasized by the distinct scoring of different surgical approaches.

## Discussion

Surgical approaches to the MTR are classified into three groups. Depending on the surface through which the exposure is made, direct exposure is provided by approaches based on the superior, lateral, and posterior aspects (Table 1). Each technique provides direct access to various structures within the MTR, including the hippocampus and amygdala (Table 1). The approach chosen depends on the specific condition being treated as well as the surgeon’s personal preference and level of experience. Although lobectomy operations are done for MTR lesions, there are many studies on selective surgical approaches in the literature. One of these, the transsylvian approach, is a superior approach frequently used for MTR pathologies since 1973 when it was developed by Yasargil [8]. The transsylvian approach is done through the floor of the sylvian fissure close to the anterior inferior portion of the inferior peri-insular sulcus of the insula. It allows for resection of the MTR without neocortical structure resection and temporal lobe retraction [1, 6] (Fig. 1B). Niemeyer introduced the selective amygdalohippocampectomy in 1958; this approach was originally called the transventricular amygdalohippocampectomy. He used a middle temporal cortical incision through the middle temporal gyrus to reach the hippocampus [20], comprising a lateral approach through any sulcus or gyrus of the lateral area of the temporal lobe. If posterior enlargement is necessary, the hippocampus and parahippocampal gyrus can be resected with subpial aspiration; however, homonymous hemianopsia is more prevalent with the transcortical approach than with the transsylvian approach [20, 21]. The sulcus between the superior and middle temporal gyrus is typically utilized for the trans-sulcal approach. The sulci endings nearest the temporal horn of the lateral ventricle require minimal resection of the white matter. Nevertheless, during sulcal dissection, the pia mater and, consequently, the underlying cortex are damaged. Moreover, it may be necessary to coagulate small sulcal veins. This procedure carries the risk of indirectly impairing cortical function [22, 23].

Hori and colleagues developed the subtemporal approach which enables the surgeon to manipulate MTR without damaging the vein of Labb´e. This method can minimize postoperative parenchymal damage [24], but a statistically significant relationship to memory loss was found on long-term follow-up. In addition, long-term follow-up revealed a statistically significant relationship between alcohol consumption and memory loss [25]. Ture and Pamir have recommended the small petrosal approach over the subtemporal approach to increase the chance of preserving the basal vessels and allowing greater surgical exposure or angle [26]. In the presigmoid-retrolabyrinthine approach, variations in venous anatomy can make retracting the basal surface of the temporal lobe difficult or impossible. In the presigmoid- retrolabyrinthine approach, the petrosal bone is drilled to obtain broad exposure of the skull-base structures. It has the benefits of minimizing brain retraction, shortening the working distance, and allowing access to critical neurovascular structures from multiple angles. It also requires less retractile force than alternative posterior techniques. However, the complexity of this technique necessitates a lengthy learning curve [27, 28]. The supratentorial-infraoccipital approach, defined by Smith and Spetzler [27], begins with gentle retraction of the occipital lobe, and the brain retractor is removed after the cerebrospinal fluid has been drained. The vein of Galen and its tributaries remain medially, allowing a surgical corridor. Disadvantages of this approach are dural sinus injury, delayed dural sinus thrombosis, and visual defects. There may also be difficulty reaching the amygdala [27, 29, 30].

The supracerebellar transtentorial approach is an effective, less-invasive avenue. Voigt and Yasargil first used this approach to remove a cavernous angioma in the posterior hippocampus [31]. Subsequently, Yonekawa and colleagues used it to clip the distal segment of a posterior cerebral artery aneurysm [32]. Moftakhar and colleagues also described using this approach for the posterior portion of the MTR [33]. Further, Türe used this approach to reach the entire MTR and has popularized it for selective amygdalohippocampectomy [3]. The risk of injury to the visual cortex and optic radiation is minimal, and it provides a safe corridor for surgical access to the middle and posterior MTR portions. Although there is more than one bridging vein, these are directly visualized in the supracerebellar transtentorial approach. In some cases, access to the MTR may be difficult with this approach, as the venous system may obstruct the superior portion of the posterior portion. In particular, the lateral cerebellar bridging veins, which may be parallel to the MRI sections, cannot be seen on the preoperative MRI.

**Fig. 5:**
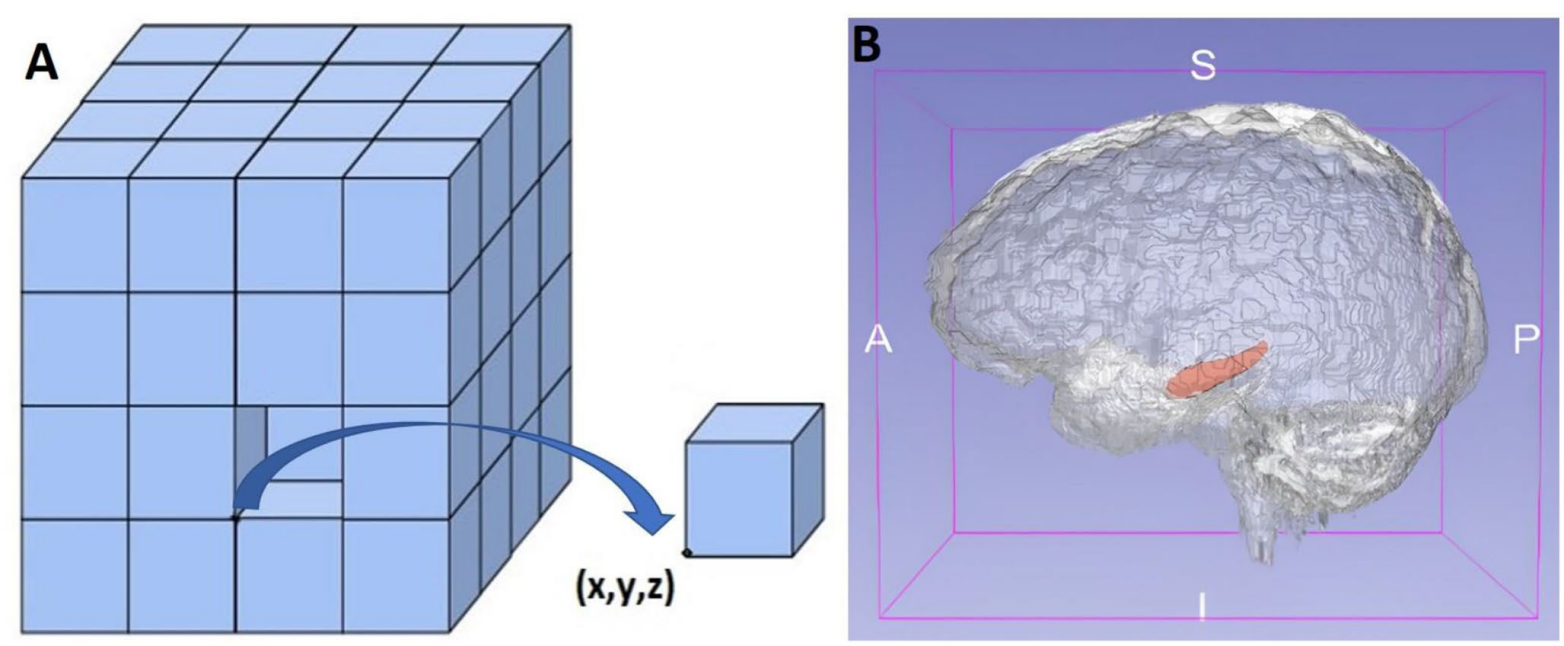
The cubic-coordinate system (x,y,z) for AI calculation. (**A**) The coordinate system represents a cubic graph as a set of wireframes composed of cubes. To utilize the path-finding algorithms on segmentation images, the images must be converted into graphical representations. Anatomical function can be added as another coordinate. (**B**) Placing MRI scans into the cubic coordinate system to match the brain parenchyma with this system can reveal the concept of digital anatomy and act as a basis for systems that can be developed and controlled

Presently, these surgical approaches are used frequently and successfully. Nevertheless, selecting an effective surgical approach to the MTR remains difficult. The complex functional anatomical structure and deep location of the MTR have been the focus of many articles regarding the success of selected techniques [3, 21, 23, 25]. Today, technological advances facilitate safer access to localized deep brain lesions similar to those in the MTR. One of these advances, MRI sequences, allow noninvasive preoperative evaluation and are increasingly used for surgical planning and strategy [34, 35]. These MRI images are also useful during surgery, along with neuronavigation devices. Neuronavigation systems function by matching the spatial positions of digital data (MRI or computed tomography) obtained from imaging studies to the surgical field. The images obtained before surgery are matched with the patient’s reference points on the operating table. Therefore, in the image, the intracranial point the surgeon is at during the operation is controlled. This technique significantly decreases morbidity and mortality when combined with neuroanatomical structure-based surgical techniques that have been used for decades. Image-guided neuronavigation systems allow more minimally invasive surgery [9]. In our study, a system to evaluate neuroimages and navigation was developed. First, MRI sequences taken from the EDEN2020 Human Brain MRI Datasets were studied [13, 14]. The BrainSuite tool was used to divide T1-weighted MRI sequences into approximately 139 major regions using an atlas-based segmentation technique. Afterward, the existing images were automatically segmented by selecting them on the 3D Slicer tool (Fig. 2). Free, open-source software was used for registration purposes [17]. This software is used to visualize image-guided procedures and for processing, segmentation, planning, and navigation [14, 16]. Thus, tract systems, arteries, and veins could be labeled with the coordinate system along their routes [10]. The pixel and voxel values of the anatomical point, as well as its anatomical structure, were labeled [15, 36] (Table 2). This method provides the benefit of trading in a cubic system with matrices (Fig. 5). MRI images obtained from the data set were used to validate the system. Subsequently, preoperative images from the Radiology Department were processed using angle and thickness sequences from previous datasets. This step is important as it allows the possibility of a personalized preoperative evaluation of patients. The 294 anatomical areas labeled and defined on 3D MRI sequences were classified as target and non-violated areas (Table 2). The target area was determined as the medial portion of the MTR. The areas detailed in Tables 1 and 2 were taken into consideration as areas not to be violated. The QL algorithm (using the Python programming language) was used to find new pathways.

AI algorithms have evolved on two basic mechanisms classified as unsupervised and supervised. In unsupervised AI applications, the aim is not to establish a cause-and-effect relationship. In this method, the aim is to find similarities and differences in a complex and large data set. In supervised machine training applications, AI has a training and development process. In this training process outputs are given to the computer and the computer software learns the relationship between outputs [10].

Machine learning (ML) is a subfield of AI that focuses on the development of algorithms and statistical models that enable computers to learn from and make predictions based on data. ML algorithms use patterns and insights from data to train models that can be used to make predictions or take actions in real-world applications. ML has a growing presence in the field of neurosurgery, where it is used to improve patient outcomes, speed up diagnosis and treatment, and support surgical planning and decision-making. Some of the applications of ML in neurosurgery include image analysis, predictive modeling, surgical planning, real-time guidance, and clinical decision support. Overall, ML has the potential to significantly improve the efficiency of neurosurgical procedures and patient outcomes [10, 37]. Reinforcement learning (RL) is a type of machine learning in which an agent learns to make decisions by performing actions in an environment to maximize a reward signal, evolved from the main group of supervised learning. The agent interacts with the environment in a sequence of trials and, in each trial, it chooses an action based on its current state, performs the action, and receives a reward signal that tells it how well the action was performed. Over time, the agent learns to map states to actions that lead to high rewards, which forms its policy [10, 18, 38]. Previously generated cubic brain parenchyma was run through the coordinate system in our study. Its target is the MTR, and the locations that should not be touched on the way to this area have been determined. An analysis of approximately 12 trillion points was used to compare the optimal linear pathways. Subsequently, the entrance points to these linear pathways were used in the QL algorithm to find nonlinear pathways similar to the selected standard pathways. Finally, the risk scores and Q-learning scores for these new pathways were listed.

In our image-guided, AI-assisted approach to risk analysis, the Q-learning score of the supracerebellar-transtentorial approach (i.e., the penalty score) was the lowest. According to Q Learning, the MTR can be reached through this method with minimal surgical damage. The trans-sulcal, subtemporal, and supratentorial-infraoccipital approaches followed (Table 3). The transsylvian approach yielded a Q-learning score of 28.30 when the middle cerebral artery and the sylvian veins were omitted, and the system was re-executed without considering them. This value was determined to be third behind the supracerebellar-transtentorial and trans-sulcal values.

In our study, the Q-learning score for the supracerebellar transtentorial approach was the highest among the examined approaches. Of interest, the total risk score of all points with pathways drawn by the reviewers differed from the total risk scores of the pathways formed and followed by QL.

## Limitations of the study

The most important limitation of this study is that the image analysis is not in real-time; all algorithm calculations were done with preoperative images. The deficit from the lack of real-time data appears, for example, with the significant retraction required of the convex temporal fossa, especially during the subtemporal approach. The literature shows that some approaches cause temporal atrophy, apparent in long-term postoperative follow-up [5]. Likewise, the occipital lobe must be retracted during the occipital interhemispheric transtentorial approach. The excessive cortical retraction required for surgical exposure may cause visual field loss or language impairment in the dominant hemisphere [39].

Another serious limitation was that certain venous structures could not be introduced into the QL algorithm. Important anatomical structures that affect the surgical outcome include the basal temporal veins in the lateral approaches and bridging veins in the posterior approaches [2]. There are insufficient studies to assess the bridging vessels in the cerebellum through neuroimaging [29], and these veins could not be incorporated into the QL algorithm. Therefore, it was surprising that the subtemporal approach received the best score. During various surgical approaches, damage to certain bundles of fibers or vascular structures is ignored. The absence of clinical symptoms or the inability to detect minor symptoms after surgery appears to have diminished the significance of these interrupted anatomical structures [1]. Advanced imaging methods, such as 7T MR, can provide more detailed data. Not incorporating temporal lobe retractions into the computation is one of our limitations. Considering variable approach diameters based on the surgical trajectory, which is significant in tissue resection is a future work for our study.

Recent advancements in MRI techniques and MR tractography, as detailed in prior studies, offer potential solutions to some of these imaging limitations, providing more comprehensive insights into the complex anatomy and white matter pathways of the brain [40]. Another limitation is the type of AI algorithm chosen. QL was used, which is a popular RL algorithm. Other algorithms, such as Aggressive aplus, Dijkstra’s Shortest Path Algorithm, and Aplus, for example, can be utilized for the same purpose. As there are no studies on the effectiveness of any of these, it is not known whether the ideal algorithm was chosen. In this initial study, AI was used to compare approaches using existing data. The addition of similar studies in which detailed data is obtained from long-term follow-up will be more effective. It is not difficult for AI to collect large amounts of operative data from multicenter study groups and to evaluate anatomical structures with relative importance and minimal clinical signs. Our research remains superficial in this regard. On the other hand, AI does not exclude data. During surgery, concurrent registration and risk assessment may provide more sensitive information.For future endeavors, there’s a pressing need to prioritize the use of expansive, standardized datasets. This approach will not only enhance the accuracy of results but also ensure that outcomes are more reflective of diverse patient populations and surgical scenarios.

## Conclusions

The number of articles regarding AI and neurosurgery is rapidly increasing [41, 42]. Six common and well-known AI data processing techniques were compared and contrasted. The preoperative images were labeled with cranial MRI voxel values on a three-dimensional coordinate plane. Arteries, veins, fiber bundles, and functional structures are important in planning the surgical strategy for neurosurgeons, and in addition to these, labeling can be done based on cisternal anatomy.

In our study, based on preoperative data, the paramedian supracerebellar transtentorial, supratentorial-infraoccipital, and subtemporal-transparahippocampal approaches to the MTR received the highest scores from QL calculations. These calculations will eventually need to be updated simultaneously with the surgical steps in real time.In data-driven AI, the more specific the data, the more useful it will be for making decisions and producing actions. With AI, surgical experiences, anatomical structures, connections, and functions can be correlated and enhanced by observing the comprehensive picture.

In the near future, it is likely that international clinical data will be recorded on the digital brain module and evaluated with AI. Moreover, during surgery, the surgeon’s use of bipolar and aspiratorlike equipment can be recorded for dataset. These data can then be used in the surgical simulation training of residents. It is likely that AI systems will make neurosurgical operations more controlled, supported, and, therefore, more effective.

## Data Availability

No datasets were generated or analysed during the current study.
